# Vitiligo and the Role of Newer Therapeutic Modalities

**DOI:** 10.7759/cureus.31022

**Published:** 2022-11-02

**Authors:** Prathmesh Nimkar, Anil Wanjari

**Affiliations:** 1 Department of Medicine, Jawaharlal Nehru Medical College, Datta Meghe Institute of Medical Science (Deemed to be University), Wardha, IND

**Keywords:** autoimmune, phototherapy, melanocyte, therapeutic modalities, vitiligo

## Abstract

Our understanding of the etiology of vitiligo, which is now categorically recognized as an autoimmune illness characterized by the occurrence of chalky-white patches over the skin as a consequence of focal melanocyte loss, has made significant strides in recent years. The notion that vitiligo results from a mix of etiologic factors that affect melanocyte functionality rather than solely due to underlying mutations, melanocytes reacting to chemical or radiation exposure, or hyperreactive T cells, has undoubtedly remained consistent. Since then, new research has contributed to our understanding of gradual depigmentation. The next stage of vitiligo research-the expansion of efficient therapeutic modalities-will be propelled by knowledge of the relative significance of such etiologic aspects and a thorough evaluation of the most targetable pathways. Although vitiligo is frequently written off as a cosmetic issue, it can have terrible psychological implications and significantly interfere with daily activities. A patient's interpersonal and social conduct may be impacted by their perception of stigmatization, which ultimately raises their chance of developing depression. This review is a summary of various theories of the pathogenesis of vitiligo as well as an overview of the therapeutic modalities that are currently available for the same.

## Introduction and background

**Figure 1:**
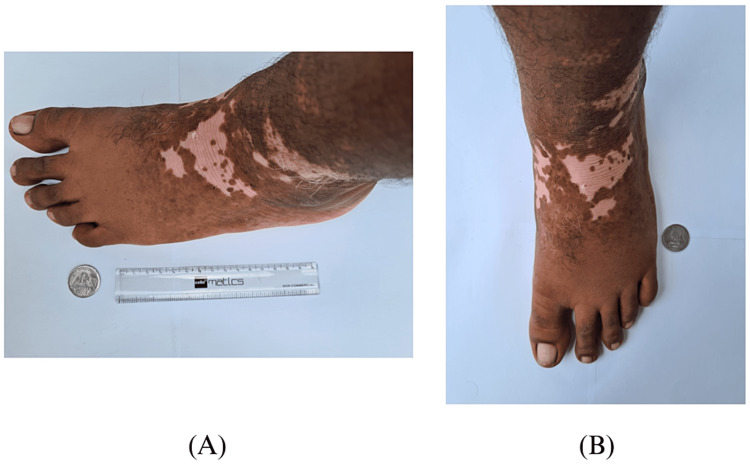
Segmental vitiligo on the left foot of a 22-year-old male patient. An Indian 1 rupee coin and a 15 cm ruler are kept for reference. Image credits: Prathmesh Nimkar, Anil Wanjari.

One of the most well-known acquired autoimmune disorders, vitiligo causes melanocyte loss in the skin and mucosa, which results in depigmentation. Depigmentation can progress throughout an individual's life, particularly in the case of generalized vitiligo. Patients with vitiligo, particularly those with darker complexions, have a significantly reduced quality of life and significant negative consequences for their sense of self-worth and social life. With an overall frequency of 0.5% to 2% in adults and children globally, vitiligo is the most prevalent depigmented skin condition [1]. Both genders are equally impacted, albeit female patients typically seek advice more often, maybe due to the higher negative societal impact than for men and boys [2, 3]. Almost a quarter of vitiligo sufferers experience the condition before the age of 10, 50% before the age of 20, and 70% to 80% before the age of 30 [4, 5]. An international consensus in 2011 recognized non-segmental vitiligo (NSV) and segmental vitiligo (SV) as the two main types of the condition. It was decided to use the term "vitiligo" to refer to all non-segmental vitiligo types. One of the consensus's most crucial choices was separating segmental vitiligo from other forms, especially due to the prognostic consequences [1, 6]. Figure 1 shows areas of depigmentation in a patient suffering from segmental vitiligo.

## Review

Pathogenesis

There are several theories as to how melanocytes are destroyed in vitiligo. Among these are genetic elements, autoimmune responses, oxidative stress, the production of inflammatory mediators, and processes for the destruction of melanocytes. It appears that both the innate and adaptive immune systems are at work. Even after the general agreement on the autoimmune character of the disease, not even a single one of these hypothesized models is sufficient in and of itself to elucidate numerous types of vitiligo. Multiple processes, such as immunological attack or cell deterioration and detachment, may be involved in the gradual loss of melanocytes [1, 7-11].

A complicated condition with both hereditary and environmental influences, vitiligo acts like a normal polygenic disease from a genetic perspective, with each particular genetic component having very little individual contribution. Nonetheless, compared to most other complex characteristics, vitiligo polygenicity is quite low, and heritability is rather high. Consequently, vitiligo's genetic basis has proven to be simpler to identify and comprehend than that of the majority of other complicated characteristics. Thus, vitiligo offers a highly amenable model for research into the genetic architecture of complicated diseases and offers crucial insights for the individualized, predictive treatment of complex disorders [12].

Oxidative stress may be crucial in triggering the immunological reactions that follow vitiligo [13]. Stressed melanocytes produce damage-associated molecular patterns (DAMPs) or autoantigens, which then trigger innate and adaptive immunity, resulting in melanocyte dysfunction and death through an inflammatory cascade. Reactive oxygen species (ROS) are stimulated by a variety of factors, and as antioxidant defenses are impaired, melanocyte redox homeostasis is lost [11], eventually causing vitiligo. The pathophysiology of vitiligo has been linked to oxidative stress and autoimmunity with genetic vulnerability, although it is unclear exactly how these pathways interact [8].

Oxidative stress and adaptive immunity in vitiligo are connected by innate immunity. It is believed that external or intrinsic stress signals generated by melanocytes and potentially keratinocytes trigger the recruitment of innate immunity early in the course of vitiligo [14, 15, 16]. Genomic expression investigation of patients' skin has revealed unusually elevated innate immunity, especially natural killer cells, in the immediate milieu of melanocytes. It has been discovered that natural killer cells invade clinically normal skin in vitiligo patients, indicating that these cells are quick to react when melanocytes are under stress [16]. In an animal model of the illness, inducible heat shock protein 70 has been demonstrated to play a crucial role in the development of the disease by causing dendritic cells to present T cells in lymphoid tissues with melanocyte-specific antigens [17]. This has been the crucial connection between natural and acquired immunity, causing melanocyte death [18, 19]. Recently, it was shown that vitiligo patches in Sinclair swine may be re-pigmented by an altered form of inducible heat shock protein 70. This finding paves the way for a potential new therapy for patients with vitiligo[20, 21].

Vitiligo's pathophysiology is also hypothesized to be influenced by both aberrant humoral and cell-mediated immune responses. Melanocytes are destroyed by cytotoxic CD8+ T lymphocytes, which particularly attack melanocytes [1]. These autoreactive T lymphocytes can detect perturbed melanocytes thanks to cytokines released by the skin. This is likely significant since dynamic processes are needed to detect melanocytes effectively in the epidermis, which is not vascularized [22]. The skin and blood of vitiligo patients, as well as a mouse model, exhibit elevated levels of interferon-gamma and interferon-gamma-induced chemokines (CXCL9 and CXCL10) [23, 24]. In a mouse model of the condition, interferon-gamma and CXCL10 are furthermore necessary for the development and persistence of the illness [23, 25]. Recently, a different study showed that serum CXCL10 levels were not just greater in vitiligo patients than in healthy controls but also associated with disease activity and reduced considerably following an effective therapeutic intervention, indicating it may be used as a biomarker to track the progression of the condition and the effectiveness of the therapeutic options [24].

Psychosocial impact of vitiligo

Among groups, vitiligo has significantly different psychosocial effects. Compared to pale-skinned people in northern Europe, the consequences are more obvious on the Indian subcontinent. Individuals with vitiligo are frequently stigmatized since depigmentation is a feature of leprosy as well. Since vitiligo can have a significant emotional weight, psychological assistance is crucial in order to set reasonable expectations for potential therapeutic modalities [26]. Along with the shame associated with having vitiligo, individuals felt guilty about the stigma that other family members who were not affected by the condition had to endure. Patients with vitiligo were reported to have lower self-esteem in prior research [27]. However, those who successfully managed their disorder were found to have better self-esteem [28].

One of the primary motivations for seeking medical help was the worry that the illness would spread and affect the entire body. Young individuals were particularly affected by the psychological effects of schooling, marriage, and employment. In elderly patients, the social cost of vitiligo in the family, which younger family members who are unaffected must bear, was a significant issue. Patients with vitiligo are frequently unable to obtain certain jobs due to their condition. On the other hand, vitiligo that appeared after securing work had less of an impact. Marriage and the challenges of getting married are areas of major impact. In some cases, vitiligo continued to be a problem even after marriage, which made it impossible to get along with in-laws, have sexual interactions, or even lead to divorce. Patients often had to deal with unwanted advice and bothersome inquiries from colleagues, relatives, and well-wishers [29].

The circumstances of the patient's life, their social support system, and the attitudes of co-workers and family members are all significant factors to take into account when assessing the psychological effects of vitiligo. Even a "minor" condition can cause the patient significant suffering. Cognitive behavioral therapy is one psychological intervention that helps individuals with vitiligo improve their body image, self-esteem, and quality of life. It also appears to have a favorable impact on the disease's progression [30]. Support and self-help groups can aid individuals in coping with this emotionally and socially damaging illness, in addition to individual therapy [29].

Therapeutic modalities

Treating vitiligo remains one of the toughest dermatological challenges to this day. Recognizing that vitiligo is not only a cosmetic condition and that there are efficient and reliable therapies is a crucial first step in managing the condition [31]. These therapies, which also include surgery, topical and systemic immunosuppressive agents, and phototherapy, may all work together to slow the progression of the condition, stabilize depigmented lesions, and promote re-pigmentation [32, 33]. The subcategory of the illness, its scope and spread, the individual's age, impact on the standard of living, and desire for therapy all play a role in the treatment plan that is ultimately chosen. Therapy works best on the face, neck, trunk, and mid-extremities, whereas the lips and distal extremities are less responsive [34]. Re-pigmentation first develops at the edges of the lesions or in a perifollicular pattern. The treatment must be administered for at least two to three months to assess its effectiveness. The most popular therapeutic modality for vitiligo, ultraviolet (UV) light-based therapy, has a better prognosis when paired with another therapy [33].

Since the majority of treatment choices are time-consuming and require protracted follow-up, management necessitates an individualized therapeutic strategy in which patients should always be consulted. Individuals with lesions affecting exposed regions should be advised on cosmetic concealment by a cosmetician or a trained nurse. These include self-tanning preparations using dihydroxyacetone, which offer long-lasting color for up to several days, and foundation-based cosmetics [1].

Since the 1950s, topical corticosteroids (TCS) have been utilized for their ability to reduce inflammation and modulate the immune system. There have been no studies determining the ideal time frame for topical corticosteroid therapy. Some authors advise using it every day for two to three months, while others advise using an intermittent strategy [35]. Topical calcineurin inhibitors (TCIs) and topical corticosteroids are often used as standard therapies for some types of vitiligo. Topical calcineurin inhibitors are typically used twice daily [36]. As per a recent meta-analysis, the therapeutic benefits of topical calcineurin inhibitors coupled with phototherapy were greater, supporting the synergic benefits of this combination treatment. When phototherapy is not an option, topical calcineurin inhibitor monotherapy may be helpful for treating neck and face lesions, especially in youngsters. A different meta-analysis found that, with the exception of the neck and face, adding topical calcineurin inhibitors to narrow-band ultraviolet B (NB-UVB) therapy did not result in substantially better outcomes as opposed to narrow-band ultraviolet B monotherapy [37].

Seeing as narrow-band ultraviolet B is safe for all age groups, it has become the primary therapeutic option for patients with vitiligo, affecting more than 10% of the body surface area [1]. Psoralen and ultraviolet A (PUVA) phototherapy are efficacious, but there are a number of drawbacks, such as phototoxic side effects, nausea, and the propensity for skin cancer. Furthermore, due to the systemic usage of psoralen and ultraviolet A, phototherapy cannot be used on youngsters or pregnant individuals. Narrow-band ultraviolet B phototherapy has increasingly replaced psoralen and ultraviolet A phototherapy since it was originally shown to be helpful for treating vitiligo in 1997. Narrow-band ultraviolet B has several advantages over psoralen and ultraviolet A, including the absence of a photosensitizer, a lower overall dosage, and fewer side effects. Narrow-band ultraviolet B even outperformed psoralen and ultraviolet A in terms of effectiveness. Other side effects of narrow-band ultraviolet B phototherapy include erythema, itching, and minor burning or discomfort; these are often tolerable and go away on their own after a few hours of treatment. As a result, narrow-band ultraviolet B phototherapy is currently regarded as the gold standard therapeutic strategy for generalized vitiligo, whereas psoralen and ultraviolet A phototherapy are still taken into consideration in some situations, like spreading vitiligo cases with deeper ultraviolet A penetration. Also, since phototherapy typically takes a long time, it is important to reassure and motivate patients to get the best possible results [38].

**Figure 2:**
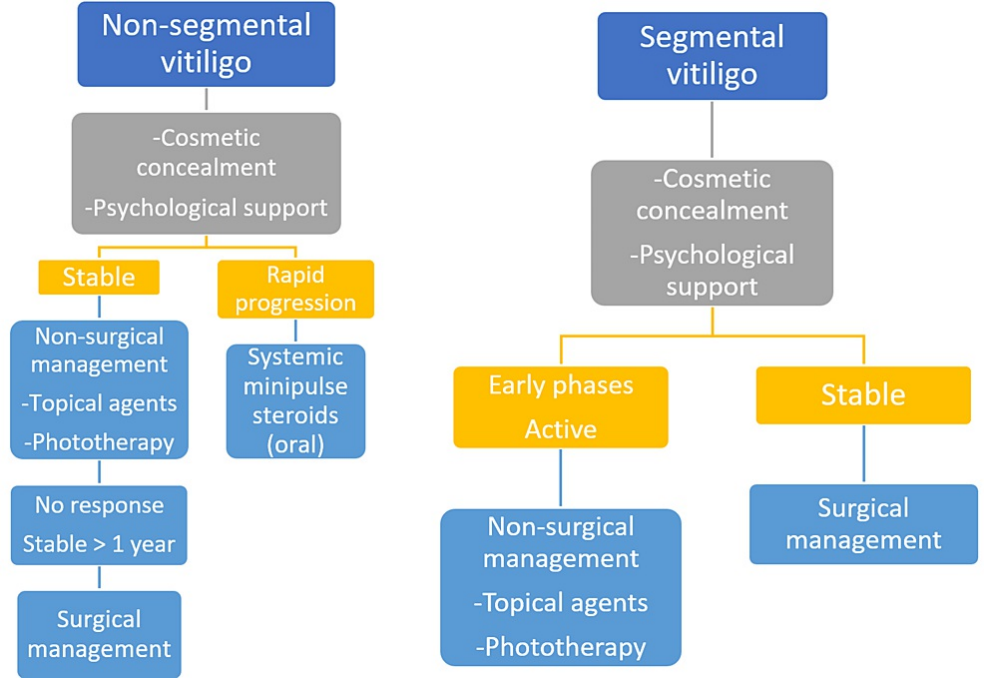
Therapeutic management of vitiligo. Image credits: Prathmesh Nimkar, Anil Wanjari.

In the previous few decades, various improvements to the currently employed surgical vitiligo treatment strategies have been made alongside the introduction of newer ground-breaking approaches. Surgical procedures can be recommended as a possible choice of treatment for individuals with segmental vitiligo as well as those with non-segmental vitiligo who have a stable illness if there is no response to medicinal therapies even after one year. The goal of the transplant is to deliver a reservoir of functional melanocytes to the affected skin so they can proliferate and migrate into regions of depigmentation [1, 39]. There are two basic surgical approaches to treating vitiligo: one uses tissue grafts and the other uses cellular grafts [35]. The therapeutic procedure involving tissue grafts involves extracting a piece of skin from the body part with the correct pigmentation, which is then transferred to the recipient location that has already been prepared without further treatment with chemicals or enzymes. A variety of instruments, namely dermabraders and other kinds of lasers, can be employed for this purpose. Suction blistering and micro-needling are further options [40, 41]. In turn, the need to treat cells beforehand ensures that the cells acquired as a consequence of the transforming method may later be employed as a graft material and applied to the damaged location, which gives cellular grafts their specificity [40]. Dermatosurgery is often seen to be the most fruitful for segmental vitiligo. This is mostly because the alterations seen in segmental vitiligo are often stable, and this parameter is seen to be the most significant for a successful surgical procedure [42, 43]. Individuals with stable non-segmental vitiligo may benefit from such treatment if combined with other current therapeutic options [35, 44]. Hence, the entire course of treatment should be enhanced with narrow-band ultraviolet B at 311 nm or psoralen and ultraviolet A phototherapy in order to augment the therapeutic outcomes acquired after the conclusion of surgical interventions [45]. Figure 2 describes an algorithm for the therapeutic management of segmental as well as non-segmental vitiligo.

The use of Janus kinase inhibitors in the management of vitiligo has shown potential [46, 47]. Janus kinase inhibitors have been demonstrated to decrease interferon-gamma signaling, which aids in the re-pigmentation of vitiligo patients. The three Janus kinase inhibitors used to treat vitiligo most frequently reported for use are tofacitinib, ruxolitinib, and baricitinib [48]. Ruxolitinib's mode of action may entail an interference with interferon-gamma signaling and Janus kinases, as evidenced by the quick improvements seen in individuals with vitiligo and alopecia areata who received treatment for the conditions. Thus, the pathogenesis of vitiligo may be influenced by the involvement of interferon-gamma and CD8+ T cell-dependent cytokine activity, which is believed to be involved in alopecia areata [23, 49, 50]. Certain investigations have discovered that ruxolitinib suppresses the function of human dendritic cells while also blocking interferon-gamma. In vivo, this decreased the production of several important cell responses thought to have a pathogenic role in vitiligo [51]. A selective Janus kinase 1 and Janus kinase 3 inhibitor called tofacitinib has been authorized for the treatment of several autoimmune conditions like rheumatoid arthritis [49]. Tofacitinib has demonstrated effectiveness in both oral and topical formulations [52, 53]. Suction blister sampling was used to assess changes in the autoimmune responses of 10 vitiligo patients receiving tofacitinib treatment. Only half of the 10 patients who got tofacitinib treatment saw re-pigmentation, which happened only in regions that received phototherapy [54]. Tofacitinib's employment in the management of vitiligo is not presently the subject of any registered clinical studies; thus, more investigation is required to ascertain the safety, effectiveness, and function of phototherapy when used in conjunction with tofacitinib [48]. Improvement with baricitinib has only been documented in one case report [49]. When a 67-year-old male vitiligo patient switched from using tofacitinib to baricitinib for the treatment of rheumatoid arthritis, he experienced total re-pigmentation [55]. All the various therapeutic modalities discussed above have been listed in Table 1 along with their examples.

**Table 1:** A summary of therapeutic modalities for vitiligo and their examples

Therapeutic modality	Examples
Immunosuppression/Immunomodulation	Topical corticosteroids (also anti-inflammatory) and topical calcineurin inhibitors
Phototherapy	Psoralen and UVA (PUVA.), excimer lamps, and narrow-band ultraviolet B (NB-UVB) lamps
Surgical treatment	Grafts: tissue grafts (mini punch, suction blister, split-thickness skin grafts), cellular grafts (cultured melanocyte and epidermal grafts)
Janus kinase inhibitors	Tofacitinib, ruxolitinib, and baricitinib

Therapeutic alternatives

The employment of systemic immunosuppressants apart from corticosteroids in vitiligo management is not well studied [1]. In certain patients, especially those with difficult-to-treat regions, oral cyclophosphamide was demonstrated to produce re-pigmentation; nevertheless, substantially adverse effects were also seen [56]. Several investigations have indicated that anti-tumor necrosis factor alpha (anti-TNF-α) medications do not show any improvements and may instead trigger the onset and progression of the disorder, as opposed to certain authors stating otherwise. [57, 58].

Platelet-rich plasma (PRP) is an autologous preparation of concentrated plasma containing a variety of growth factors. These growth factors could hypothetically stimulate melanocytes [59]. Nevertheless, treating vitiligo with platelet-rich plasma alone might not be successful. According to a study, platelet-rich plasma and narrow-band ultraviolet B combined for therapy yielded superior outcomes over narrow-band ultraviolet B alone [60]. Hence, platelet-rich plasma can cure vitiligo more effectively when used in conjunction with other therapies. However, since these trials combined a better therapeutic modality with a small intervention, physicians should exercise caution when interpreting the findings [61]. Additionally, many injections spaced closely apart are an unpleasant procedure and can cause koebnerization [62]. These results need to be confirmed by broader, longer-follow-up randomized controlled trials.

Numerous medicines containing antioxidant enzymes have been utilized to treat vitiligo due to the role that oxidative stress plays in the etiology of the condition. Although there is a strong case for utilizing topical antioxidants to treat vitiligo, studies have produced mixed outcomes. This is likely because it is challenging to transfer active antioxidants straight into the skin [1].

## Conclusions

The etiology and pathophysiology of vitiligo are still enigmas, despite significant ongoing advancements in our understanding of this complicated skin disease. Further investigation is necessary to completely comprehend the etiology of this emotionally and socially damaging disease since there are still unanswered concerns about what ultimately causes melanocyte death. Currently available therapeutic modalities include, but are not limited to, topical corticosteroids and topical calcineurin inhibitors; phototherapy (narrow-band ultraviolet B, psoralen, and ultraviolet A; excimer lamps); surgical procedures (tissue and cellular grafts); and Janus kinase inhibitors, which aim at preventing melanocyte destruction or promoting their regeneration while keeping the adverse effects to a minimum. In addition to a particular therapy, patients with vitiligo also need a psychiatric consultation and appropriate counseling. Patients' quality of life improves as a result of this since their levels of depression are reduced. To find new treatment strategies and medications that could prevent or halt the progression of vitiligo or even cure the disease, it is imperative to have a better understanding of the clinical and molecular factors that result in metabolic abnormalities and ultimately lead to autoimmunity and melanocyte loss. Increasing the comparability and usefulness of forthcoming clinical studies for vitiligo is also a crucial concern.
